# Victories of the Doctor and the Sanitarian

**Published:** 1917-11-17

**Authors:** Woods Hutchinson

**Affiliations:** Fellow of the American Academy of Medicine.


### CAMP DISEASE ON THE BATTLE FRONTS.

### Victories of the Doctor and the Sanitarian.

By Dr. WOODS HUTCHINSON, Fellow of the American Academy of Medicine.

Dr. Woods Hutchinson, on the 8th inst., delivered at the House of the Royal Society of Medicine the second Chadwick Lecture of his series on "The Part of Hygiene in the European War."

People were apt to think that the part of the doctor and the sanitarian in this war was insignificant when contrasted with that of the soldier. But it must be remembered that the real enemy of the soldier was the germs he carried with him. So great a breeder of epidemics was the old camp that the toll used to be from four to seven deaths from disease to every death directly due to enemy missiles or weapons. The position was now reversed, because there were three deaths from actual battle to every one from disease. The largest element in this saving of life was attributable to the use of vaccines, such as those against smallpox and typhoid fever, the latter having been, indeed, one of the most dramatic elements in the struggle. Neither our foes nor our Allies were as well prepared with these measures as we were, consequently there was a heavier death-rate from typhoid on other friendly fronts than on our own. During six months on the three Western fronts which he had just brought to a close, a case of typhoid fever in a military hospital was considered a rarity. And great importance had been attached by us to such simple matters as keeping the water clean, disposing of waste, and caring as much as possible for the soldier's cleanliness.

### Some Remarkable Facts.

A consequence was that the amount of disease in the trenches was less than that found in barracks in times of peace. Among the diseases which had had to be fought on the Western front was measles, which at one time caused much discomfort and disability. The troops who suffered from it most severely were the Colonials and those hailing from the Western Islands of Scotland; indeed, measles seemed to attack the men of the finest physique. Diphtheria, cerebro-spinal meningitis, and even common colds—which were contagious and infectious—also received careful attention. It was very gratifying to find that though the armies had, for three years, been in the most abominable conditions and weather, and on soil which was said to breed rheumatism, that trouble had been conspicuous by its absence. In many places on the Western front, especially in the earlier phases, there had been great difficulty in procuring sufficient water for drinking purposes. Villages had a small well, which proved ample for the needs of peace time, but the inhabitants seldom bathed, and their wants in that respect were most primitive; but the matter was very different with 10,000 men to the mile, including reserves. In many cases dams had to be built, and water conducted for the troops by means of pipes. That was especially necessary on the Italian and French fronts. The sanitary officer was a very important person on the Western front. Largely through the activities of Colonel Horrocks, a very effective chlorinating apparatus for the water on a large scale had been for some time in use. It consisted of a large lorry, with tanks and an engine upon it, and the oxygenating, chlorinating, and precipitating apparatus. Into it water was pumped out of any chance pond or mud-hole; it was then precipitated and cleared, and the chlorine injected into it. In another tank oxygen was added, and it emerged pure sparkling drinking-water. He had seen this done to water from forbidding-looking mud-holes between slag-heaps, and the apparatus could supply water fit for drinking enough for 5,000 men in twelve hours. He did not hesitate to say that the drinking-water for the soldier at the Front was better guarded than was the water consumed by civilians in ordinary peace-times. The basis of the deadly gas which the Germans commenced to use in this war—the first time in history, as he hoped it would be the last—was also chlorine, and that was, too, the essence of Dakin's solution which had proved such a powerful germicide in surgical practice. Chlorine had saved more lives during this war than, in the form of poison-gas, it had destroyed.

### Trench Fever, its Causes, Complications, and Control.

Trench fever, the exact cause of which was yet to seek, bore a considerable likeness to malaria. It was very apt to be followed by kidney complications. The authorities took up a philosophical attitude in regard to its nature, labelling it "P.U.O."—pyrexia of unknown origin. One medical officer introduced his individuality into a parallel case by labelling a condition "G.O.K.," which, it eventually transpired, meant "God only knows." Trench foot might last a considerable time, and was a very definite state, brought about by the cold and wet of the trenches. It commenced like a severe case of chilblains, accompanied by redness and a burning pain in the skin. Later it looked like frost-bite, and gangrene might supervene. Sloughing and the consequent need for amputation, however, were now seldom seen. A contributing cause in earlier stages of the war seemed to have been the tight winding of the puttees, and as these were woollen they shrank when they became wet, and the pressure on the tissues was thereby increased and the condition aggravated. The establishment of "side-walks" or duck boards, on the Canadian plan, in the trenches had greatly mitigated the abominable condition of the trenches. A further improvement followed the use of indiarubber long boots. Attention to personal comfort, to changes of underlinen, periodic hot baths, and the care of the feet made all the difference between vigorous men ready to fight anywhere, and men who were in various stages of invalidism. In these measures the steam sterilisation of the soldier's clothes while he was having his hot bath must be given a prominent place. It had been noted that the incidence of trench fever showed a curious parallelism to the degree of lice infestation.

Shell-shock Cases only 1 per Cent. of Cases Treated in England.

With regard to shell-shock, the authorities declined now to recognise as definite shell-shock any but those cases in which the man had been blown ten feet into the air, or had been buried for at least twenty minutes. In all the military hospitals in England at the present time there were only 1,400 cases of shell-shock, or 1 per cent. of the number under treatment. There were 3,000 fewer cases of lunacy in the country during 1916 than in 1913. Some unkind person had alleged that a portion of the 3,000 had been found posts in Government offices! The war had shown that the human brain and nervous system were about the toughest thing in the world. In this war were men from every clime and of every colour, yet none had stood the strain of the campaign so well as the highly civilised, citizenised soldier in the London regiments: these men showed an extraordinarily low incidence of shell-shock and mental disorder. And Mott showed that of those who did exhibit signs of breakdown 65 per cent. had a bad nervous inheritance, and would probably have broken down in any circumstances.

Venereal Diseases; Some Plain Speaking.

Venereal diseases had challenged much thought and attention. The work which was done by the Royal Commission had been carried into the Army. Much loose talk had been indulged in, but that was largely negatived by a study of the figures in regard to venereal disease. The general average for those diseases in the armies of Europe had ranged from 6 per cent. to 12 and even 20 per cent. of the total Forces; he referred to the period 1890-1905. In 1905 the percentage among troops in barracks was 12, but shortly before the war it had been reduced. The present figure for the British Army in France was $2\frac{4}{10}$ per cent., as against 6 per cent. in 1913. In the American Army the former figure was 5 per cent.: it was now about $\frac{1}{2}$ of 1 per cent. Drunkenness among soldiers in France was very exceptional, in fact the self-control and self-respect of the men in the ranks was magnificent. If only the main cause of the spread of venereal disease could be dealt with, the prostitute, the greatest step forward would be made. She was, as a rule, a poor, wretched, half-witted creature, who sold her body because she had no brains worth speaking of to sell: she should be taken charge of as a permanent ward of the State, and placed in beautiful cottage colonies in the country. If the young soldier were given opportunities to mix and dance with nice girls he would never miss the bad ones, for his mind was, as a rule, cleaner than those of some of the middle-aged who were constantly talking on the subject. And it should never be forgotten how important a factor in the spread of venereal disease was the apparently respectable owner of premises who let out his places for the accommodation of these poor women.

Dr. Woods Hutchinson concluded with the exhibition by means of the epidiascope of a striking series of photographs which he had brought back from the Western fronts.

